# Robust Real-Time Traffic Surveillance with Deep Learning

**DOI:** 10.1155/2021/4632353

**Published:** 2021-12-27

**Authors:** Jessica Fernández, José M. Cañas, Vanessa Fernández, Sergio Paniego

**Affiliations:** Universidad Rey Juan Carlos, Móstoles, Spain

## Abstract

Real-time vehicle monitoring in highways, roads, and streets may provide useful data both for infrastructure planning and for traffic management in general. Even though it is a classic research area in computer vision, advances in neural networks for object detection and classification, especially in the last years, made this area even more appealing due to the effectiveness of these methods. This study presents TrafficSensor, a system that employs deep learning techniques for automatic vehicle tracking and classification on highways using a calibrated and fixed camera. A new traffic image dataset was created to train the models, which includes real traffic images in poor lightning or weather conditions and low-resolution images. The proposed system consists mainly of two modules, first one responsible of vehicle detection and classification and a second one for vehicle tracking. For the first module, several neural models were tested and objectively compared, and finally, the YOLOv3 and YOLOv4-based network trained on the new traffic dataset were selected. The second module combines a simple spatial association algorithm with a more sophisticated KLT (Kanade–Lucas–Tomasi) tracker to follow the vehicles on the road. Several experiments have been conducted on challenging traffic videos in order to validate the system with real data. Experimental results show that the proposed system is able to successfully detect, track, and classify vehicles traveling on a highway on real time.

## 1. Introduction 

Number of vehicles on earth is increasing rapidly. According to data provided by International Organization of Motor Vehicle Manufacturers (OICA, https://www.oica.net/), the number of vehicles produced in the last years is way more than 70 million vehicles per year. This number is increasing very quickly, equally the number of travel kilometers increases even more quickly. This explosion in the number of moving vehicles raises several challenges of different types: environmental, economical, and infrastructure management. At this moment, it is clear that managing such large number of vehicles is one of the biggest problems that countries worldwide have to deal with. Classic vehicle monitoring techniques cannot deal with such huge amount of data nor make an intelligent use of it. It is clear that new sophisticated paradigms are needed to deal with this challenging task.

The main goal of intelligent transportation systems (ITSs) is to monitor the different vehicle transport networks in a smart way. For this, they make use of the different available technologies such as dedicated sensors and advanced video cameras. The objective of this monitoring is to extract useful information that can be used to coordinate the vehicle traffic networks. Eventually, by means of these systems, we want to minimize congestion and to enhance mobility.

Video cameras are the most used sensors on ITSs systems. Their simple installation and maintenance combined with their rich nature of the information make them one of the best solutions when it comes to surveillance and monitoring. Depending on the conditions of the ITSs system, it will be necessary to use moving cameras or fixed cameras. In addition to the cameras, ITSs systems traditionally made use of other sensors such as radars for speed enforcement or inductive loops and laser and infrared sensors for vehicle classification [1–6]. Systems based on these sensors try to classify the vehicles by extracting certain information such as the vehicle's length and number or distance between axles. Although they may provide a better accuracy in general, they require an intrusive installation and not all of them provide the possibility of multilane monitoring. Another drawback is their initial high installation cost, which is an important factor to take into account when comparing ITS systems. In fact, when evaluating this kind of systems, not only the initial price but also the whole system life cycle must be taken into consideration. Finally, the information provided by these traditional systems is basic and cannot be used to extract high level traffic data such as vehicle orientation, position, or other parameters that can be used for traffic law enforcement.

Usage of video cameras in traffic surveillance [7–9] typically was limited to passive monitoring tasks or very basic automated processing. The advances in image processing algorithms in the last decade specially in the deep neural networks area have opened the door to more sophisticated systems based on computer vision. Nowadays, with these advances, we can create systems not only able to detect vehicles in normal situations but with capacity to recognize and classify vehicles in very challenging situations. This may be the base to perform high level tasks such as automated traffic management, automatic incident detection, law enforcement, fog, and other weather conditions and many other incidents.

The study presents a vision-based traffic monitoring system, named TrafficSensor, that includes a robust vehicle detection and classification algorithm and a new technique for dealing with occlusions [10–12]. It is the evolution of a previous system [13] towards a higher reliability and good performance even in challenging lightning or weather conditions, and poor camera resolution while keeping real-time operation. TrafficSensor is based on the use of a fixed camera to detect and monitor vehicles.  Section 2 (Related Works) reviews relevant studies on vehicle classification [14–18]. The system core functionality is described in the Section 3 (TrafficSensor: A Deep Learning-Based Traffic Monitoring Tool), where the details for the vehicle tracking and deep learning-based detection algorithms are presented.  Section 4 (Experimental Validation) presents several tests performed to validate the system functionality and the quantitative obtained results. Finally, Section 5 (Conclusion) summarizes the main lessons extracted from this work.

## 2. Related Works

The literature provides many publications dealing with vehicle monitoring [19–21], even recognizing the vehicle model [22]. To perform such monitoring, it is necessary to detect the vehicles and then to follow them up. A technique widely used for vehicle detection is background subtraction [23–27]. The background subtraction technique is a technique widely used to detect objects such as the difference between a current pixel and a reference pixel, called background. Huang [28] used the Gaussian mixture to detect the background and subsequently subtract it. This guarantees that the background we extract corresponds to the lighting of that moment [29]. The mixture of Gaussian (MOG) proposes to model the intensity of the pixels with a mixture of *k* Gaussian distributions. MOG is a technique that first applied to the problem of background subtraction. TrafficMonitor [13] makes use of an improved version of the proposed MOG by Zivkovic [30]. The advantage of this method is that for each pixel, the number of Gaussian to be used can be adapted. Another technique very similar to background subtraction is the absolute difference (sum of absolute differences (SAD)) between two sequences. Samhitha et al. [31] presented a technique based on the absolute difference (SAD) between two consecutive frames. Guerrero-Gomez-Olmedo et al. [32] used the histogram of oriented gradients (HOG) to detect vehicles. HOG is a type of feature descriptor. It converts the local information of the gradients for each pixel into a representation of the image that captures the global shape of the object into a feature vector.

For vehicle tracking [33–38], many solutions rely on the features. To follow-up, Wang et al. [39] employed a technique based on features called scale-invariant feature transform (SIFT) [34] and optical flow. SIFT is an algorithm used to extract characteristics from images. Optical flow is the pattern of movement of the image objects between two consecutive frames caused by the movement of the object. Mu et al. [35] also used SIFT to track vehicles. Huang and Barth [40] proposed an algorithm to carry out vehicle tracking and resolution of occlusions. In this algorithm, they use a color model based on mean-shift to identify which vehicle each 3 × 3 pixel patch belongs to when there is an occlusion. In other cases, 2D or 3D [41] models are used to do the tracking. Leotta and Mundy [42] employed this technique to detect vehicles using a deformable template that adjusts to identify different forms of vehicles. Huang [28] and Baker and Sullivan [43] used Kalman filters and Guerrero-Gómez-Olmedo et al. [32] employed extended Kalman filters (EKF) [44]. The Kalman filter is an algorithm to update, observation by observation, the linear projection of a system of variables on the set of available information, as new information becomes available. The extended Kalman filter consists of a variation of the Kalman filter to trackle the state estimation problem when the model is possibly nonlinear.

Regarding image classifiers, Vedaldi et al. [45] proposed a novel three-stage classifier, which combines linear, quasilinear, and nonlinear kernel SVMs. They showed that increasing the nonlinearity of the kernels increases their discriminative power, at the cost of an increased computational complexity. Their aim was to learn an SVM classifier [46], where rather than using a prespecified kernel, the kernel is learnt to be a linear combination of given base kernels.

One of the most heavily studied paradigms for object detection [47, 48] and classification is deep learning. The convolutional neural network (CNN) is a feed-forward type of the machine learning algorithm that have shown impressive results and robustness in visual object detection. They have been widely explored in the context of vehicle monitoring too. In Migel et al.'s work [49], the vehicle identification and classification are performed for each extracted portion of the input image, simultaneously using the designed CNN. That is, a softmax layer is used as the classifier to perform vehicle classification. Caffe [50] framework was used to benchmark the performance of the vehicle detection system. Sensa et al. [51] presented an intelligent traffic congestion detection method using the CNN. The dataset used in this experiment is the road traffic condition images from CCTV camera in Jakarta during 29 April–5 May 2017 that can be obtained from lewatmana.comhttp://lewatmana.com.

Yang et al. [52] proposed a detection method using a single image to generate the 3D space coordinate information of the object using monocular vision for autonomous driving. Their method is built by modifying the fast R-CNN using multitask learning, and thus is named multitask faster R-CNN (MT faster RCNN). For the experiments, the KITTI dataset was used.

Luo et al. [53] presented a model based on the faster RCNN with NAS optimization and feature enrichment to perform the effective detection of multiscale vehicle targets in traffic scenes. Luo et al. proposed a Retinex-based image adaptive correction algorithm (RIAC) (to reduce the influence of shadows and illumination), conducted neural architecture search (NAS) on the backbone network used for feature extraction of the faster RCNN (to generate the optimal cross-layer connection to extract multilayer features more effectively), and used the object feature enrichment that combines the multilayer feature information and the context information of the last layer after cross-layer connection (to enrich the information of vehicle targets and improve the robustness of the model for challenging targets such as small scale and severe occlusion). Their model has been trained and tested on the UN-DETRAC dataset.

Redmon et al. [54] presented YOLO, a new approach to object detection. YOLO reframes object detection as a single regression problem, straight from image pixels to bounding box coordinates and class probabilities. Use You Only Look Once (YOLO) at an image to predict what objects are present and where they are. Jean-Francois Rajotte et al. [55] did automatic annotations that were performed with the YOLO detector. Kwan et al. [56] used YOLOv1 to detect vehicle in real time.

Mahto et al. [57] used the object detection algorithm YOLOv4 and optimized it for vehicle detection. To improve YOLOv4, they proposed optimize the anchor box using k-means clustering (ABK), the nonmaximum suppression with distance-IoU (DIoU-NMS), the spatial attention module (Sam), and the self-adversarial training (SAT). The UA-DETRAC Benchmark dataset was used to train and test the method.

Zhang et al. [58] proposed an improved RetinaNet. Their algorithm uses octave convolution instead of the traditional convolution layer and a weighted feature pyramid network (WFPN) structure to limit the propagation of gradients between different levels. To evaluate the result, the DETRAC dataset was used.

Szegedy et al. [59] presented a network that is based on the convolutional DNN defined by [60]. It consists of total 7 layers, the first 5 of which being convolutional and the last 2 fully connected. Each layer uses a rectified linear unit as a nonlinear transformation. Three of the convolutional layers have in addition max pooling.

## 3. TrafficSensor: A Deep Learning-Based Traffic Monitoring Tool

**Figure 1:**
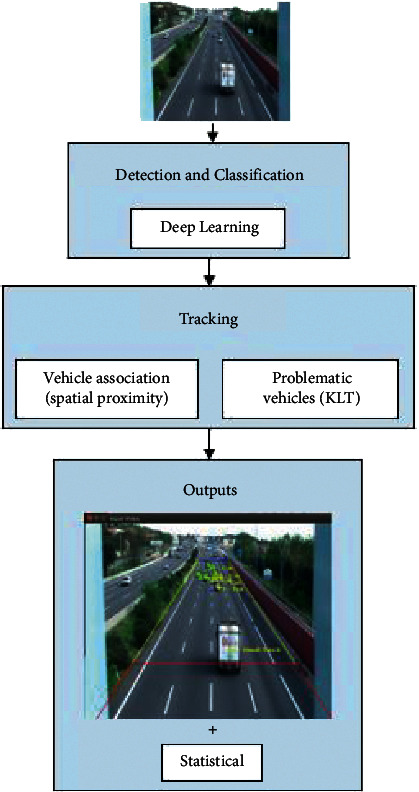
Block diagram of the TrafficSensor system.

TrafficSensor tool is able to monitor traffic in real time and classify the vehicles into 7 categories: motorcycles, cars, vans, buses, trucks, small trucks, and tank trucks. It consists of three main blocks: vehicles detection, vehicles classification, and vehicles tracking, as shown in Figure 1. They are implemented in two separate modules, as detections and their classification are carried out jointly because deep learning is used. The tracking focuses on spatial proximity, and if it fails, KLT is used. All detected blobs will be tracked over time.

**Figure 2:**
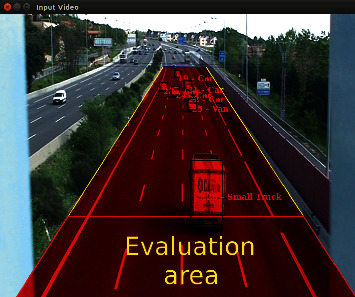
Evaluation area.

There is a single image area where detection, classification, and tracking are carried out. This area, that is called evaluation area, is marked in the image by the user to identify where on the road we want to focus the detections, as shown in Figure 2. TrafficSensor is designed to monitor outgoing traffic flow, although it can be extrapolated to incoming traffic flow.

### 3.1. Deep Learning-Based Detection and Classification

The system takes input images acquired from the video being monitored. These images pass as input to the neural network, where various vehicles are detected and classified. All information is stored at each moment, so that it can be tracked based on the information recorded from the previous moment. TrafficSensor supports trained neural networks with different neural frameworks (TensorFlow, Darknet, and Keras) in order to detect and classify the different vehicles that appear in the image.

**Figure 3:**
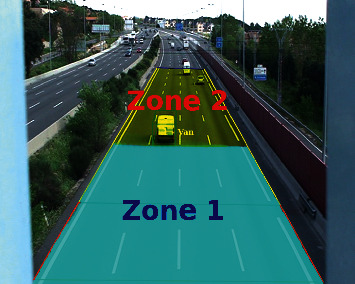
Evaluation zones.

In the detection and classification block, the system implements these criteria:

- Inside the evaluation area, there are two zones (Figure 3). The zone 1 matches with the half of the evaluation area where the vehicles enter. In this zone, it is easier to detect and classify the vehicles because they are bigger than that in other areas of the image. The zone 2 refers to the half through which vehicles leave the evaluation zone. This zone is more complex, since the vehicles have smaller size than in the zone 1.
- Vehicles always enter in the evaluation area through zone 1. They can never appear suddenly. For this reason, no new vehicle can appear in the middle of the road. A new vehicle can never be detected in zone 2.
- If a vehicle is not detected in zone 1 during five-frame sequence, it will be a false positive. It will be discarded.
- Any vehicle that is in zone 2 will be considered a correct vehicle. If the vehicle is not detected with deep learning, KLT will be used to locate it.

Three different frameworks (TensorFlow, Keras, and Darknet) and four neural network models have been tested in order to evaluate which one is better for the final TrafficSensor application. Specifically, the SSD MobileNetV2 network with TensorFlow, the SSD VGG-16 network with Keras, and YOLOv3 and YOLOv4 with Darknet.

#### 3.1.1. SSD MobileNetV2 Network

**Figure 4:**
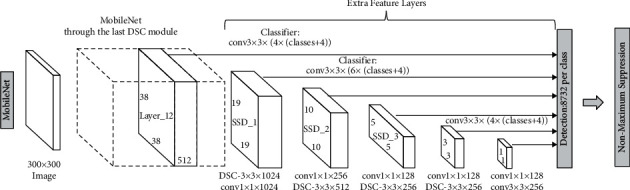
SSD MobileNet V2 network.

The SSD MobileNetV2 network (Figure 4) used was trained with the COCO dataset. To use this network, we utilized the configuration file ssd mobilenet v2 coco.config.

This network is formed by a SSD and a MobileNet V2. MobileNet V2 gets the maps of features to perform the classification and detection in the subsequent layers. The SSD approach is based on a convolutional feed-forward network that produces a set of bounding boxes fixed in size and punctuates the presence of object class instances in those bounding boxes. After this, it carries out nonmaximum suppression to produce the final detections.

#### 3.1.2. SSD VGG-16 Network

**Figure 5:**
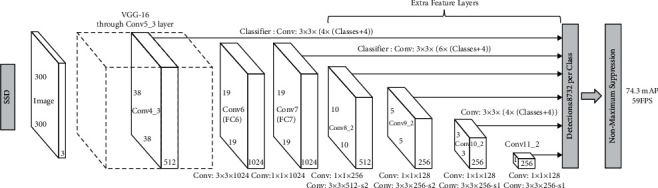
SSD network model.

**Figure 6:**
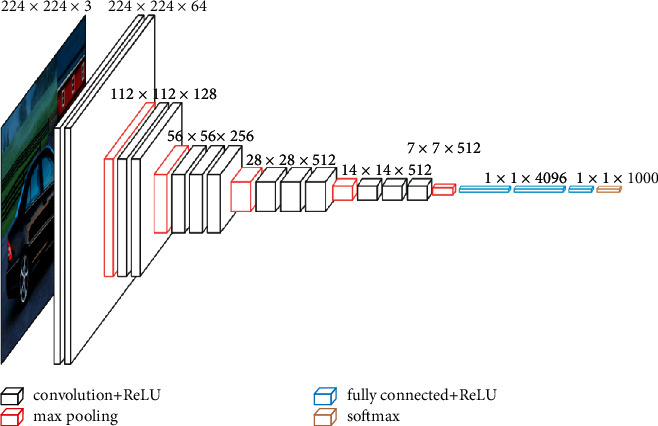
VGG-16 model.

Another SSD network has been used with VGG-16 as its base network, pretrained with ImageNet.  Figure 5 shows this network model. VGG-16 consists of 16 layers, of which 13 are convolutional layers, 2 fully connected layers, and a softmax layer that is used to classify.  Figure 6 shows how the architecture of the VGG-16 network is.

#### 3.1.3. YOLOv3

You Only Look Once (YOLO) imposes strong spatial constraints on bounding box predictions, since each cell in the grid only predicts *N* bounding boxes (*N* being a fixed parameter) and can only have one class. This spatial restriction limits the number of nearby objects that our model can predict.

**Figure 7:**
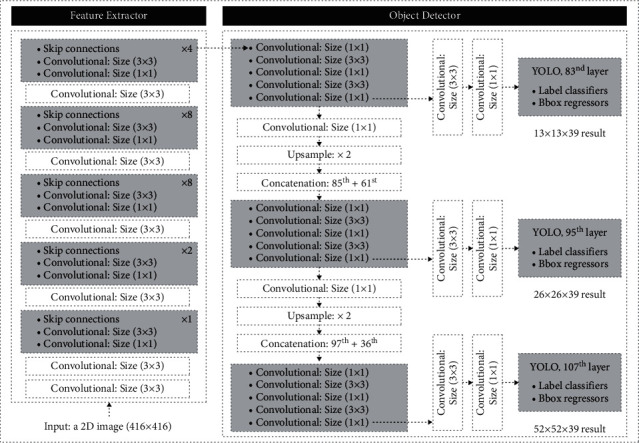
YOLOv3 model.

**Figure 8:**
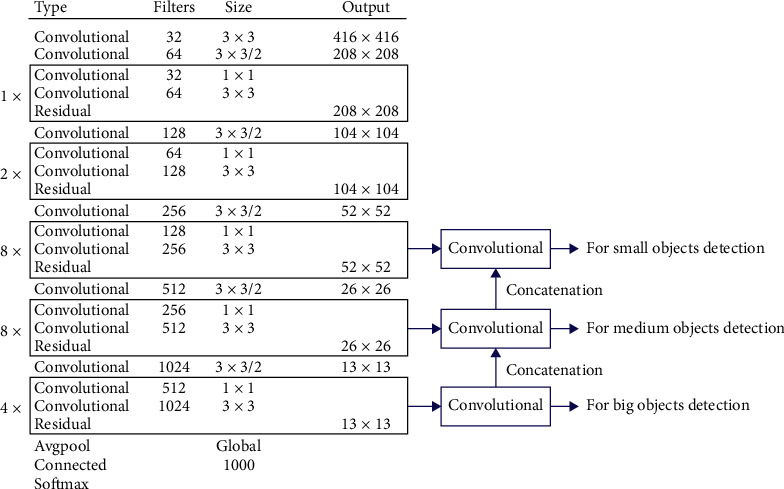
Darknet-53 model.

The YOLOv3 [61] network (Figure 7) is made up of a total of 107 layers, which can be grouped into two groups, one in charge of extracting features and another in charge of detecting objects:

- Feature extraction (from layers 1 to 75): it is the Darknet-53 network trained with ImageNet, which is composed of 53 convolutional layers (Figure 8). This network has 416 × 416 × 3 images as input and features as output 3D 13 × 13 × 1024 and incorporates 23 residual layers. When a neural network increases in depth its precision when it comes to propagating the characteristics, it tends to degrade, leading to a greater error in training. The residual layers are used to solve this problem.
- Objects detection (from layers 76 to 107): it takes the 3D features (13 × 13 × 1024) as input and with that performs object detection. The uniqueness of this network lies in its ability to detect objects on three different scales, making it a very powerful network before the change of scale. To do this, it extracts characteristics on three different scales (13 × 13 × 39, 26 × 26 × 39, and 52 × 52 × 39). These characteristics pass to the final YOLO layer, which classifies the object label with class logistic regressions and locates objects with bounding boxes regressors.

#### 3.1.4. YOLOv4

**Figure 9:**
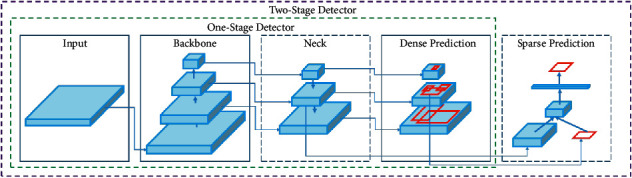
YOLOv4 object detector.

YOLOv4 [62] is the fourth iteration of the famous YOLO architecture that continues improving the previous versions with the latest advances introduced in the literature. It consists of 3 main components: backbone, neck, and head (Figure 9). For the backbone, it uses CSPDarknet53 [63], for the neck, SSP [64] and PAN [65], and YOLOv3 [61] for the head.

This network allows real-time object detection on a conventional GPU, thanks to its improvements on speed comparing it to other approaches and even its previous versions.

### 3.2. Vehicle Tracking

**Figure 10:**
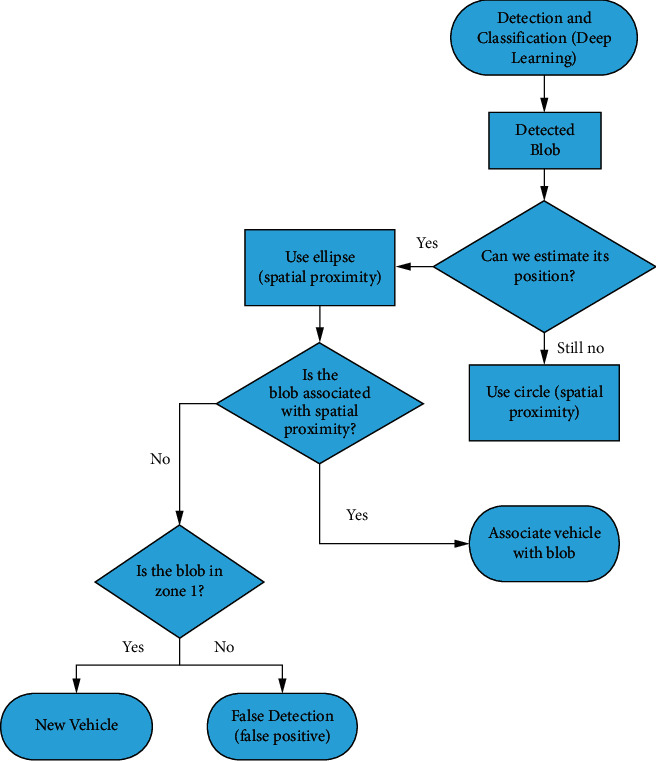
Execution flowchart of detected blobs.

**Figure 11:**
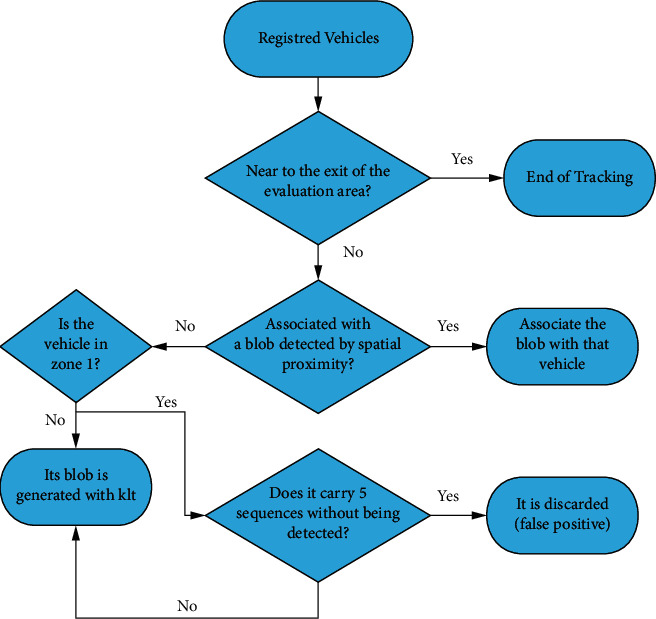
Flowchart of registered vehicles.

The execution flow of the tracking module is shown in Figure 10, which shows the steps when a new blob is detected inside the image, and in Figure 11, which illustrates the procedure that is carried out on already registered vehicles. The tracking focuses on associating the current detections with the vehicles stored at the previous instant. Several points are considered:

- If a vehicle arrives at the end of the evaluation area, it will be removed from tracking
- The vehicles stored of the instant (*t* − 1) are examined in order to pair them with the vehicles detected at the instant (*t*). This pairing will be carried out between the vehicles in (*t*) and (*t* − 1), which have the least Euclidean distance between their centers.
- If the vehicle *t* associated with the vehicle (*t* − 1) is not within the circular or elliptical area around the center of the vehicle (*t* − 1), it will not be matched to it
- If through space proximity we are not able to pair a (*t* − 1) vehicle, we will use KLT

Spatial proximity and KLT algorithm are used to perform the vehicle tracking. Spatial proximity works fine for separate vehicles, but in real videos, it is very likely that we have occlusions and vehicles that are quite complex to detect, especially when they are small in the image because they are far from the camera. The feature-based tracking algorithm KLT is used then, and it is a good complement to the deep learning robustness.

#### 3.2.1. Spatial Proximity Tracking

Typically, the difference of pixels in the image between the position of a vehicle in (*t* − 1) and in (*t*) is very small. Therefore, a vehicle in (*t*) will be in an area very close to that same vehicle in (*t* − 1). When we search for a vehicle in (*t*), we should find it in a small circular radius around the position of that same vehicle in (*t* − 1). Spatial proximity tracking in TrafficSensor is based on [13]. It estimates the area where you should locate a vehicle based on its position in (*t* − 1). As the vehicles move forward, this area will be updated.

At first, the area is taken as a circle because the system does not have enough data about its orientation. But as the vehicle advances, the system has enough information to know its orientation, and so, it takes the area as an ellipse whose center corresponds to the center of the vehicle in (*t* − 1). It is considered that we have enough information to estimate its orientation when we have the position of the vehicle in 6 frames. Linear regression is used to calculate the orientation of the vehicle based on the position the vehicle will take as it progresses. Once we have information about the orientation, we will define the search area as an ellipse whose center is the same as the vehicle in *t* − 1 and direction calculated with the following equation.

(1) $$\begin{array}{c} \rho \left(r_{i}\right)=2\left(\sqrt{1+\frac{r^{2}}{2}}-1\right). \end{array}$$

**Figure 12:**
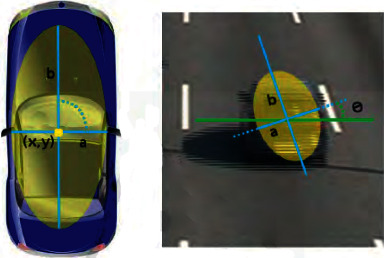
Vehicle-associated 2D ellipse.

The pairings between the vehicles detected at time (*t*) and the vehicles stored from time (*t* − 1) are limited to vehicles that fall within the area of the circle or the ellipse that is obtained based on the position of the vehicle at time (*t* − 1). The ellipses are defined as *C*_*xc*,*yc*,*ω*_, where *ω* is the orientation and (*x*_*c*_, *y*_*c*_) is the center of the vehicle. These parameters are shown in Figure 12.

**Figure 13:**
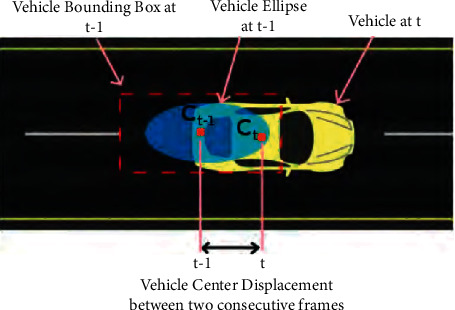
Proximity tracking ellipse.

The 2D vehicle, whose center is *B*(*x*, *y*), will be inside the ellipse *C*_*xc*,*yc*,*ω*_, if it accomplishes the following equations:

(2) $$\begin{array}{c} C_{\omega }=\arctan\left(\frac{a_{x}}{a_{y}}\right), \end{array}$$

(3) $$\begin{array}{c} {\left(\frac{\cos\left(C_{\omega }\right)\left(B_{x}-C_{x_{c}}\right)+\sin\left(C_{\omega }\right)\left(B_{y}-C_{y_{c}}\right)}{b}\right)}^{2}+ \end{array}$$

where *a*_*x*_ and *a*_*y*_ are the components of the orientation vector.  Figure 13 shows the tracking between two consecutive vehicles.

**Figure 14:**
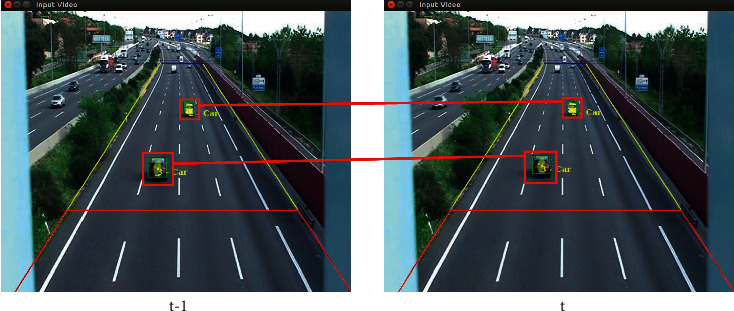
Tracking with spatial proximity TrafficSensor.

Figure 14 shows an example of TrafficSensor where the tracking of two vehicles by space proximity is shown. The vehicle identified as 2 in the image of the instant (*t* − 1) is associated with the closest vehicle to its position in the current image (*t*).

A detection must be within a certain area around the blob detected in (*t* − 1) to be identified as the same vehicle. It could happen in the case that two vehicles will fall into that area. Therefore, it is necessary to take into account the Euclidean distance between the center of the blob of the instant (*t* − 1) and the center of the blob in (*t*). The blob of the instant (*t*) that is at a smaller distance from the blob of the instant (*t* − 1) and of course within the area around blob (*t* − 1) will be considered the same vehicle than that of (*t* − 1). That is to say, if this is true, the blobs of (*t* − 1) and (*t*) correspond to the same vehicle but in consecutive moments.

#### 3.2.2. KLT Tracking

The follow-up is mainly based on spatial proximity, but KLT will be used in problematic cases, thus making our system more robust. KLT will be calculated in all sequences to update the feature points. If a vehicle is not detected either because there is an occlusion or it is very far away, KLT will be used, as it has proven to work well even in occlusions during a small number of consecutive frames.

**Figure 15:**
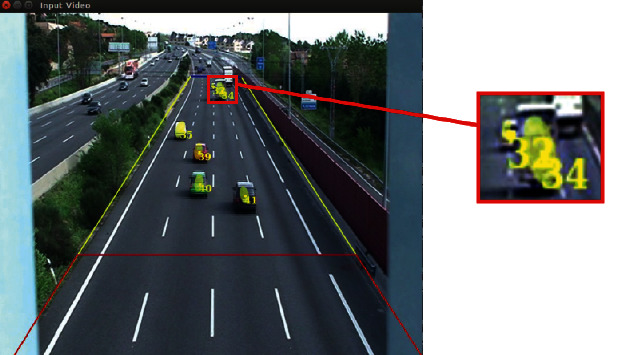
Tracking with KLT in TrafficSensor.

To use KLT, we need to know the center of mass of the vehicles and their visual features. Depending on the feature points of the vehicle in (*t* − 1), KLT calculates the matching for each feature point and as a result generates a new set of feature points corresponding to the vehicle in question. In order to achieve a correct match, the system is based on votes of the feature points that an object has associated.  Figure 15 shows an example.

KLT is a feature tracking algorithm [? ? ]. KLT is a differential and local method in which the neighborhood of each pixel is analyzed. The algorithm assumes that the optical flow is constant in a neighborhood. The equation of the optical flow is solved for all the pixels in this neighborhood by the method of least squares. For the calculation of the velocity vectors, the following formula is used:

(4) $$\begin{array}{c} \left[\begin{array}{c} u\\ v \end{array}\right]={\left[\begin{array}{cc} \sum_{i}I_{xi}^{2} & \sum_{i}I_{xi}I_{yi}\\ \sum_{i}I_{xi}I_{yi} & \sum_{i}I_{yi}^{2} \end{array}\right]}^{-1}\left[\begin{array}{c} -\sum_{i}I_{xi}I_{ti}\\ -\sum_{i}I_{yi}I_{ti} \end{array}\right]. \end{array}$$

The vector (*u*, *v*) is the displacement vector of the optical flow. *I*_*x*_ is the mean of gradient in *x* between two consecutive images, that is, if *I*(*t*) is the image of the instant current and *I*(*t*+1) is the image at the next instant, *I*_*x*_ of these frames is

(5) $$\begin{array}{c} I_{x}=\frac{I_{x}\left(t\right)+I_{x}\left(t+1\right)}{2}, \end{array}$$

where *I*_*x*_(*t*) is the gradient in the *x* axis of the image *I*(*t*) and *I*_*x*_(*t*+1) is the gradient in *x* of the image *I*(*t*+1). *I*_*y*_ is the mean of the gradients in *y* of the image *I*(*t*) and *I*(*t*+1):

(6) $$\begin{array}{c} I_{y}=\frac{I_{y}\left(t\right)+I_{y}\left(t+1\right)}{2}. \end{array}$$

It is the difference between *I*(*t*) smoothed and *I*(*t*+1) smoothed:

(7) $$\begin{array}{c} I_{t}=I^{\prime }\left(t+1\right)-I^{\prime }\left(t\right). \end{array}$$

KLT is applied in the form of kernels of size *ω* × *ω* throughout from image. The size of the kernels must be defined according to the amount of movement that the image has. A small kernel would be ideal for evaluating small displacements of a point. Using a large kernel increases the risk of getting an error, but there are cases where the displacement of a point is very big and this is necessary.

**Figure 16:**
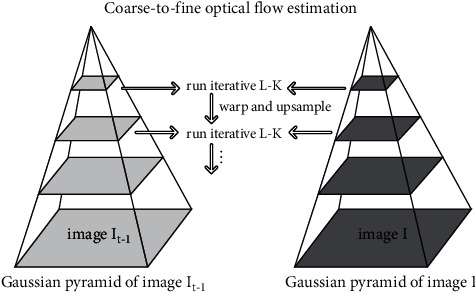
Pyramidal KLT.

TrafficSensor uses the pyramidal implementation [66], which Jean-Yves Bouguet introduced. On it, the KLT algorithm is applied recursively over an image pyramid, as shown in Figure 16.

## 4. Experimental Validation

The proposed system has been validated with a dataset of real traffic images, which has been divided into training and test subsets. In addition, the 4 studied neural networks for the detection and classification module of TrafficSensor have been quantitatively compared using an open source tool, named DetectionMetrics, so the best one could be selected for the final system. This measuring tool is publicly available and was created as a part of this work, but it is generic and usable in any other visual detection application. In addition, the final system was tested and validated both with good lightning images and, in particular, with poor images or images in bad weather conditions, which are typically present in real deployments.

### 4.1. Dataset

To train and evaluate the networks, a new dataset was created. This dataset includes images in good weather conditions, images in bad weather conditions (with fog and rain), and poor quality images. This dataset consists of the following:

- The database built by Redouane Kachach in his doctoral thesis [13]. That database consists of 3460 good quality images.
- The GRAM Road-Traffic Monitoring (GRAM-RTM) database created by Guerrero-Gomez-Olmedo et al. [32]. This database is made up of images extracted from three videos. The first video, called M-30 (7520 frames), was recorded on a sunny day. The second, called M-30-HD (9390 frames), was recorded in a similar location but during a cloudy day. The third, called Urban1 (23435 frames), was recorded at a busy intersection. From this large database, 3646 images of the M-30-HD video and 1348 of the M-30 video were used.
- Images were collected from open online cameras. 615 were about rain situations and 705 of poor quality images.

In total, the dataset for TrafficSensor consists of 9774 images. All of them have been manually tagged with the labelImg tool https://github.com/tzutalin/labelImg, using 7 possible classes: car, motorcycle, van, bus, truck, small truck, and tank truck. In these 9774 images, we have a total of 48914 samples distributed, as given in Table 1.

**Figure 17:**
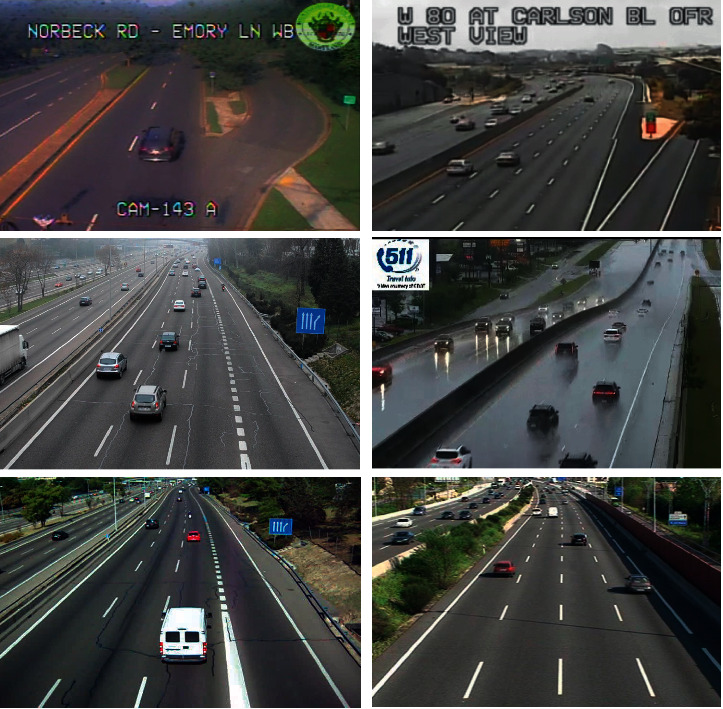
TrafficSensor dataset samples.

Table 2 provides the number of images that exist for each type of image (good conditions, bad weather, and poor quality), and Figure 17 shows some illustrative images of our database.

Of these 9774 images, one part was used in training and another in the test.  Table 3 provides the distribution of images according to training and test.

For the training of the involved neural networks, the training database was divided itself into train and validation subsets. Out of the 9246 images, 7401 were used as train and 1845 as validation.  Table 4 provides the number of images that has been used in training depending on its type (good quality, poor quality, and bad weather).

### 4.2. DetectionMetrics Tool

DetectionMetrics (https://jderobot.github.io/DetectionMetrics/) is an opensource research software application that has been created and used to quantitatively evaluate the performance of pretrained neural networks and our visual traffic surveillance application.

It provides a toolbox of utilities oriented to simplify the development and testing of solutions based on visual object detection. The application comes with a GUI (based on Qt) and can also be used through command line. It is designed to generate experiment results from running a set of neural networks models over many datasets. Currently, it comes with the following utilities: viewer, detector, evaluator, deployer, labelling, and converter.

**Figure 18:**
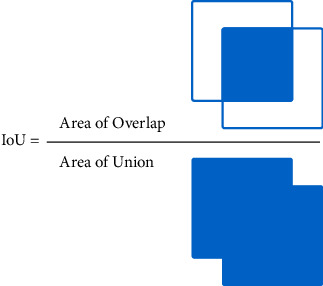
IoU formula.

It comprises a generic infrastructure to evaluate object detection algorithms against a dataset and calculate common statistics:

(i) IntersectionOverUnion (IoU) measures the accuracy of a detection in a particular dataset and follows the following formula:
  (8) $$\begin{array}{c} \text{IoU}=\frac{\text{AreaofOverlap}}{\text{AreaofUnion}}. \end{array}$$
Here, AreaofOverlap is the area that belongs to the intersection between prediction and ground truth, while AreaofUnion is the sum area (without repetition of the overlap) of the prediction and ground truth as shown in Figure 18.
(ii) Precision is the total correct detections among the number of detections obtained. The precision of DetectionMetrics is the average (mean average precision (mAP)) for those predictions that have an IoU greater than a threshold (0.5).
  (9) $$\begin{array}{c} \text{Precision}=\frac{\text{TP}}{\text{TP}+\text{FP}}. \end{array}$$
(iii) Recall is the number of correct detections among the number of actual detections, that is, ground truth detections. Like precision, averaging (mean average recall (mAR)) of detections having a higher IoU is obtained to 0.5.

This tool is compatible with Linux, Windows, and MacOS because it is provided as a Docker image in addition to the common source code installation. It allows to evaluate models trained in TensorFlow, PyTorch, Keras, Caffe, and Darknet, and it supports the most common dataset formats in object detection (YOLO, COCO, ImageNet, and Pascal VOC) and can use different image input sources (videos and webcam).

The main workflow used for the experiments is called headless evaluation. This workflow involves mainly two of the tools included in DetectionMetrics: detector and evaluator. In this mode, a researcher determines a set of experiments that will run independently and unattended, retrieving a final report with the previous described objective metrics. DetectionMetrics receives a batch of datasets and deep learning pretrained models, predicts the objects on the images using each model over each dataset, and outputs the report with metrics of the performance for each scenario.

#### 4.2.1. Detector

Detector generates a new annotated dataset with the predicted labels given a pretrained neural network model and a dataset. This new generated dataset contains the images from the datasets along with the detected objects, their position, and the level of confidence for the predictions. It supports the most common deep learning frameworks: TensorFlow, Keras, Darknet, Caffe, and PyTorch.

During the detection process, DetectionMetrics shows the predictions using viewer, showing the ground truth and predictions on the image while running. This is very convenient as qualitative feedback about the network performance.

#### 4.2.2. Evaluator

Evaluator receives two datasets with the same format as input, one considered to be the ground truth and the other the generated detections dataset, and retrieves an evaluation report with metrics for every experiment, showing how each network was performed over each dataset. It supports both mAP and mAR metrics.

### 4.3. Comparison of Neural Networks

The four trained networks with 3 different neural frameworks were tested (SSD MobilenetV2, SSD VGG-16, and YOLOv3 and YOLOv4) with the images of good conditions. YOLOv3 and YOLOv4 have also been tested with the weights prior to training with our database. This experiment was performed on a GeForce RTX 3070 graphics card, whose main features are given in Table 5.

The quantitative results obtained in the experiment are given in Table 6. Those of the YOLO networks are better than those of SSD MobilenetV2 and SSD VGG-16. Looking at the detection times, it can be seen that all the trained networks show similar speeds. With the pretrained weights, the detection times are bigger because these weights have obtained training with more classes. The achieved quality results (mAP and mAR) with the pretrained weights are worse than with the trained weights as expected. This makes clear the need to retrain the network with an adequate database that adjusts the network model to the data we want to detect.

As given, YOLOv4 further improves both the detection quality and speed over YOLOv3. YOLOv4 uses data augmentation. It interprets the same information from different points of view. YOLOv4 is based on pixel-by-pixel modifications in the training images (color changes, texture, black or white patches, cuts, and other modifications) that help the algorithm to increase its precision and flexibility, but without affecting its performance in terms of speed. In the performance achieved in the tests, the speed of YOLOv4 versus YOLOv3 has increased by 13%. The result is practically equal to that indicated by the authors of YOLOv4 [62], who reported a speed increase of 12%.

### 4.4. Experimental Validation in Good Lightning Conditions

For the final TrafficSensor application, YOLOv3 and YOLOv4 were the selected networks, as they obtained the best results. To validate these final networks, the quality of the whole system has been measured with DetectionMetrics and the created testing dataset. For the sake of comparison, the quality of the initial base-line system, named TrafficMonitor [13] and without deep learning layers, has also been evaluated with the same dataset and measuring tool. In addition, TrafficSensor has also been compared to Deep SORT (Simple, Online and Realtime Tracking with a Deep Association Metric) [67], which is an algorithm commonly used in object tracking. It is an extension to SORT (Simple, Online and Realtime Tracker) [68] that incorporates appearance information through a pretrained association metric. All systems were evaluated with the same good condition videos and images.

The results obtained are given in Table 7. YOLOv4 and YOLOv3 have similar results although YOLOv4 is slightly better. This result was expected as the authors of YOLOv4 [62] indicated that the quality of the detections was superior to that of YOLOv3.

The results of TrafficSensor outperform those of TrafficMonitor. In the successive tests with TrafficMonitor, we have appreciated that it does not work well with distant vehicles (in many cases cars are classified how motorcycles), and it has difficulty to differentiate between car and van. The small vans are confused with cars. This is because the classification is done using 3D models; for this reason, a small 3D van model can be closer to the 3D model of a car than to that of a large van.

In Deep SORT, the YOLOv3 Darknet network trained with our dataset has been used, and the results obtained by TrafficSensor and Deep SORT are very similar. TrafficSensor performs slightly better because it predicts the position of vehicles when they are not detected. Deep SORT uses the Kalman filter to predict and track, but predictions are used to improve detections, not to predict if there is no detection.

### 4.5. Experimental Validation in Poor Conditions

**Figure 19:**
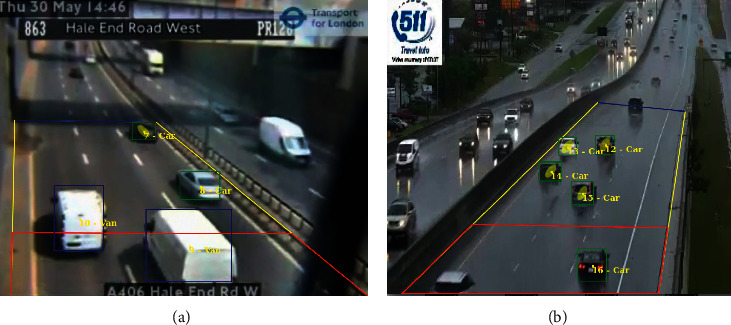
TrafficSensor with poor resolution (a) and bad weather (b) videos.

The final TrafficSensor system was also evaluated with bad weather conditions and poor quality videos, as shown in Figure 19. Tables 8 and 9 provide the obtained experimental results.

Despite being in rainy conditions, the system is able to work successfully and with very good results. In this test, it can be seen that TrafficMonitor is not so robust because it is not able to function correctly with rain. In the case of Deep SORT, again the results are similar to TrafficSensor.

With all the experimental results gathered, it can be said that TrafficSensor is robust against poor quality images and bad weather conditions. In addition, it is able to continue tracking vehicles when they are far away from the camera. Obviously, it works better with nearby vehicles, as there they are easier to detect, but it is still able to detect and track the distant ones with great quality.

Comparing the experimental results in the videos, the performance with poor quality videos and unfavorable weather conditions is slightly better than for good quality videos. This can be explained since the minimum requirements we set for good quality images are higher than those for bad weather conditions and poor-quality videos. We do not expect the system to be able to detect distant vehicles in bad weather conditions and poor quality videos. It is not even easy for humans to classify such vehicles. The images in the dataset have been labelled following this approach.

When evaluating the results obtained by Deep SORT, they are similar to those of TrafficSensor. TrafficSensor has greater precision since in cases where the neural network is not capable of detecting, it predicts such detection using the tracking algorithm.

### 4.6. Processing times

In the TrafficSensor system, three main processes can be identified: image processing (obtaining images, displaying images, and obtaining data from the delimited road), detection, and tracking. Their computing time performance, both with YOLOv3 and YOLOv4, has been monitored and evaluated.  Table 10 provides the obtained results.

## 5. Conclusion

TrafficSensor system is a solution for vehicle surveillance using deep learning. It is based on a previous nondeep learning solution, named TrafficMonitor [13]. The old solution was based on volumetric 3D patterns, SVM for vehicle classification and background subtraction. This system was able to distinguish between 5 possible classes (motorcycles, cars, vans, buses, and trucks). All these steps were replaced by a neural network for detection and classification. Four state-of-the-art network models have been experimentally tested, even coming from different neural frameworks (Keras, TensorFlow, and Darknet) and with different types of images. The proposed deep learning system classifies the vehicles based on 7 classes: motorcycles, cars, vans, buses, small trucks, trucks, and tank trucks.

A new dataset was created to train and evaluate the new system, including a variety of images such as poor quality images or adverse weather conditions besides the typical good lightning images. TrafficSensor has proven to be robust to bad weather conditions, blurred or low resolution traffic images. This improvement was achieved, thanks to training with the new extensive dataset and the combination of spatial correspondence tracking and KLT tracking on the deep learning-based detections.

Both the YOLOv3 and YOLOv4 networks have been selected for TrafficSensor for their great results. Although, YOLOv4 obtains better results in terms of quality and speed than YOLOv3.

In addition, a new opensource tool has been created to quantitatively and automatically measure the quality of several neural networks for the visual detection task using large datasets. It supports the most widely used neural frameworks (PyTorch, TensorFlow, Keras, and Darknet) and the most common dataset formats in object detection (YOLO, COCO, ImageNet, and Pascal VOC). It measures some useful detection statistics such as IntersectionOverUnion, precision, recall, and inference times. It is publicly available.

As future lines, we intend to test more new state-of-the-art network models for visual object detection, to extend the custom dataset with more images of bad quality or bad lightning conditions, of incoming traffic flow, and to explore the use of attention-based models. In addition, we plan to use DetectionMetrics tool in the medical images domain.

## Figures and Tables

**Table 1:** Database samples.

Class	Sample
Car	38976
Motorcycle	1886
Van	5631
Bus	401
Truck	963
Small truck	938
Tank truck	119

**Table 2:** Database images.

	*N* of images
Good conditions	8406
Bad weather	663
Poor quality	705

**Table 3:** Dataset distribution.

Type	Training images	Test images
Good conditions	6717	389
Bad weather	1892	71
Poor quality	637	68
Total	9246	528

**Table 4:** Training dataset.

Type	Training images	Validation images	Total
Good conditions	5323	1394	6717
Bad weather	1568	324	1892
Poor quality	510	127	637
Total	7401	1845	9246

**Table 5:** GeForce RTX 3070 specs.

GPU engine specs	
NVIDIA CUDA cores	5888
Base clock (GHz)	1.5
Boost clock (GHz)	1.73
Memory specs	
Memory speed	14 Gbps
Standard memory config	8 GB GDDR56
Memory interface width	256 bit
Memory bandwidth (GB/sec)	448

**Table 6:** Results of trained networks.

Neural networks	Framework	mAP	mAR	Mean inference time (ms)
ssd300adam.h5	Keras	0.7478	0.7831	13
ssd_mobilenet.pb	TensorFlow	0.5484	0.61361	10
yolov3voc.weights	Darknet	0.8926	0.9009	15
yolov3voc_pre_trained.weights	Darknet	0.4577	0.5843	34
yolov4.weights	Darknet	0.9056	0.9670	13
yolov4_pre_trained.weights	Darknet	0.4799	0.5879	24

**Table 7:** Results of good conditions video.

System	mAP	mAR
TrafficSensor YOLOv3	0.8926	0.9009
TrafficSensor YOLOv4	0.9056	0.9670
TrafficMonitor	0.4374	0.5940
Deep SORT	0.8164	0.8689

**Table 8:** Results of bad weather video.

System	mAP	mAR
TrafficSensor YOLOv3	0.9899	0.9926
TrafficSensor YOLOv4	0.9904	0.9949
TrafficMonitor	0.2407	0.3162
Deep SORT	0.9801	0.9824

**Table 9:** Results of poor quality video.

System	mAP	mAR
TrafficSensor YOLOv3	0.9439	0.9444
TrafficSensor YOLOv4	0.9902	0.9911
TrafficMonitor	0.4479	0.6303
Deep SORT	0.8852	0.8910

**Table 10:** Processing time.

Function	With YOLOv3 (ms/call)	With YOLOv4 (ms/call)
Image processing	10	10
Detection algorithm	15	13
Tracking algorithm	18	18

## Data Availability

The data used to support the findings of this study are available from the corresponding author upon request. Part of them, the GRAM Road-Traffic Monitoring database, comes from a third party source which has been properly cited [32]. In addition, the source code of the DetectionMetrics tool, which has been used for experiments, is publicly available at https://github.com/JdeRobot/DetectionMetrics.
